# Editorial: New advancement in tumor microenvironment remodeling and cancer therapy

**DOI:** 10.3389/fcell.2024.1384567

**Published:** 2024-03-07

**Authors:** Yi Yao, Ying Shen, James C. Yao, Xiangsheng Zuo

**Affiliations:** ^1^ Cancer Center, Renmin Hospital of Wuhan University, Wuhan, China; ^2^ Hubei Provincial Research Center for Precision Medicine of Cancer, Wuhan, China; ^3^ State Key Laboratory of Oncology in South China, Guangzhou, China; ^4^ Guangdong Provincial Clinical Research Center for Cancer, Guangzhou, China; ^5^ Sun Yat-sen University Cancer Center, Guangzhou, China; ^6^ Department of Gastrointestinal Medical Oncology, University of Texas MD Anderson Cancer Center, Houston, TX, United States

**Keywords:** TME, cancer therapeutics, immune check inhibitor, PD-L1, tumor mutational burden

Tumor progression and treatment processes have the potential to remodel tumor microenvironment (TME) (Benavente et al., 2020; Winkler et al., 2020; Arora and Pal, 2021). Conversely, the TME plays a substantial role in altering tumor growth, metastasis, therapeutic response, and development of therapeutic resistance (Sahai et al., 2020; Winkler et al., 2020; Wu et al., 2021; Mantovani et al., 2022). This Research Topic is dedicated to publishing original research and review articles exploring critical factors reshaping TME and the interplays between the TME remodeling and tumor progression, immune therapeutic response, and resistance.

Cancer cells evade immune surveillance through PD-1/PD-L1 axis that inhibits activation and functions of tumor-infiltrating lymphocytes (TILs) in TME. Blocking the PD-1/PD-L1 signaling has shown remarkable effectiveness in restoring T cells from exhaustion and normalizing the dysregulated TME, leading to cancer cell eradication (Cha et al., 2019). Immune checkpoint therapies (ICTs) such as the antibodies against this axis exhibit potent antitumor activities in various cancers, including lung adenocarcinoma (LUAD) (Han et al., 2020; Sun et al., 2023). Determination of PD-L1 expression is crucial for selecting patients benefiting from this therapeutic approach, as PD-L1 expression level in cancer cells is positively associated with a favorable response (Ribas and Hu-Lieskovan, 2016). The regulation of PD-L1 involves intrinsic (cancer cell-associated) and extrinsic (TME-originating) factors, including dysregulation of oncogenic signaling pathways and dependence on inflammatory signals, cytokines, and metabolites. The TME, a complex ecosystem supporting tumor growth, undergoes dynamic communication and metabolic symbiosis between tumor and non-tumor cell populations. Iron, a multifunctional micronutrient, plays a key role in signaling pathways within tumor cells and the TME, influencing cancer progression (Sacco et al., 2021). The iron addiction phenotype, driven by reprogramming intracellular iron metabolism and interactions with immune cells, has dual effects, promoting cancer growth and suppressing antitumor immune functions (Cassim and Pouyssegur, 2019). The study by Battaglia et al. revealed a significant correlation between iron density and PD-L1 expression in LUAD tissues. *In vitro* analyses of H460 and A549 LUAD cells showed increased PD-L1 mRNA and protein levels in an iron-enriched microenvironment, mediated by reactive oxygen species (ROS)/c-Myc signaling pathway; iron-induced PD-L1 overexpression inhibited T cell activity, demonstrated by reduced IFN-γ release in a co-culture system of tumor and T cells, emphasizing the impact of iron on immune modulation in LUAD. In TCGA LUAD datasets, the levels of transferrin receptor CD71, indicative of an iron-addicted phenotype, correlate with elevated PD-L1 mRNA. This study explores a novel association between high iron density and elevated PD-L1 expression in LUAD and the findings open a door for combinatorial strategies that consider TME iron levels to enhance the efficacy of anti-PD-1/PD-L1-based immune therapies for LUAD patients.

ICTs have significantly transformed clinical outcomes for cancer patients, providing enduring clinical benefits, and even leading to a cure in a subset of individuals (Sharma et al., 2023). Cancer patient response to ICTs (e.g., pembrolizumab) varies across the tumor subtypes, necessitating robust biomarkers for patient selection (Sharma et al., 2023). While PD-L1 expression and microsatellite instability-high (MSI-H) are FDA-approved indicators, tumor mutational burden (TMB), the number of somatic mutations per mega base of interrogated genomic sequence, is emerging as a promising biomarker in solid tumors (Singal et al., 2019; Sha et al., 2020). TMB varies across malignancies. The data from KEYNOTE-158 study showing TMB-high (≥10 mut/Mb) correlates with better pembrolizumab therapy outcomes in multiple cancer types, lead to FDA approval of pembrolizumab for TMB-high tumor subgroup (Marabelle et al., 2020). However, concerns arise regarding the TMB cutoff of 10 mut/Mb and its applicability beyond the KEYNOTE-158 study. The study by Mo et al. aimed to statistically determine the optimal universal cutoff for defining TMB-high in the published clinical trials, predicting anti-PD-L1 therapy efficacy in diverse advanced solid tumors. By integrating MSK-IMPACT TMB data and objective response rate (ORR) across various solid cancer types, the authors identified 10 mut/Mb as the optimal cutoff, strongly correlated with PD-L1 blockade ORR. This study provides a new universal TMB-high cutoff evidence in support of the KEYNOTE-158 study for guiding clinical decisions and addressing challenges associated with tumor-agnostic pembrolizumab approval in TMB-high cases.

Oral squamous cell carcinoma (OSCC) is a highly malignant disease with increasing incidence and lacks effective treatments, urging the exploration of new therapeutic targets. The B7 family, a group of 10 structurally related, cell-surface protein ligands, including PD-L1 (B7-H1), encoded by CD274 gene, and inducible T cell costimulatory ligand (ICOSLG, B7-H2), encoded by CD275 gene, bind to receptors on lymphocytes that regulate adaptive immune responses in cancers (Ni and Dong, 2017). PD-L1 (B7-H1) plays a crucial role in immune escape in OSCC (Zhao et al., 2023). The association of ICOSLG expression levels with immunosuppression, tumor progression and prognosis of different solid cancer types such as gastric cancer (Chen et al., 2003), colorectal cancer (Cao et al., 2018) and glioblastoma (Iwata et al., 2020) has been studied. However, the specific role of ICOSLG in OSCC remains largely unexplored. In a retrospective study, Dong et al. observed that ICOSLG was ubiquitously expressed in OSCC cancer cells, cancer-associated fibroblasts, and TILs. Elevated ICOSLG levels were found to be correlated with advanced TNM stage and lymph node metastasis, serving as a predictive factor for decreased overall or metastasis-free survival in OSCC patients. These findings underscore the potential of ICOSLG as a promising target for precision immunotherapy in the context of OSCC.

B cell malignancies, encompassing B-cell non-Hodgkin’s lymphomas (B-NHL) and B-cell chronic lymphocytic leukemias (B-CLL), are prevalent in cancers that arise in B lymphocytes. B-NHL ranks as the seventh most common cancer in the United States, with 74,000 new cases annually. Obinutuzumab, the first humanized type II glycoengineered anti-CD20 monoclonal antibody, displayed superior outcomes in clinical trials for B-NHL and B-CLL (Goede et al., 2014; Gabellier and Cartron, 2016). Despite these advancements, relapse remains common, highlighting the need to further understand the mechanisms to improve the patient therapy. Chemotherapy resistance often stems from malignant B-cell migration to the bone marrow and interaction with the stromal layer. The study by Fagnano et al. explored whether stromal cells impeded this type II anti-CD20 antibody mechanisms, contributing to therapeutic resistance by employing co-cultures of Raji or Daudi human B lymphoblastoid cells and M210B4 bone marrow stromal cells. The results showed direct contact with stromal cell inhibited obinutuzumab-induced programmed cell death, cellular phagocytosis, and cytotoxicity; stromal interference with B-cell adhesion and actin remodeling, was possibly linked to CD20 downregulation. Understanding the significance of direct interactions between stromal and tumor cells may provide great insights for developing better strategies to enhance Obinutuzumab efficacy by targeting both stromal and tumor cells and ultimately improve outcomes in B-cell malignancies.

Heterotypic 3D human tumor cell models, combining tumor cells and fibroblasts, strike a balance by mimicking solid tumor phenomena effectively (Franchi-Mendes et al., 2021). The anthracycline chemotherapy drug Doxorubicin (DOX) is used for the treatment of various cancers, including colon cancer, by disrupting tumor cell DNA to inhibit replication. However, DOX resistance hinders its effectiveness (Chen et al., 2018). Valente et al. study explored the interplay between fibroblasts, DOX resistance, and spheroid characteristics. With establishing DOX-sensitive and -resistant spheroids from HCT116 colon cancer cells with or without fibroblasts, the study unveiled that fibroblasts stabilized spheroids and altered hypoxia- and inflammation-related gene expression. DOX resistance impacted drug internalization. These findings underscore the significance of models resembling *in vivo* tumor cell interactions with TME, offering valuable insights for testing drug treatments, and understanding resistance mechanisms.

Moreover, Yang et al. identified that SH3 domain-binding glutamate-rich protein 3 (SH3BGRL3) was upregulated in acute myeloid leukemia (AML) and was negatively correlated with survival of AML patients. Furthermore, *in-vitro* studies showed that circSH3BGRL3/circRNA_0010984 promoted AML cell proliferation by inhibiting miR-375 activity and increasing YAP1 expression. The study by Jiang et al. revealed that miRNA-146a-5p expression was downregulated in gastric cancer (GC) tissues, and *in-vitro* study showed that miRNA-146a-5p inhibited GC cell growth and promoted GC cell apoptosis by directly suppressing CDC14A expression. Zhang et al. identified a set of neurotransmitter receptor-related colorectal cancer prognostic gene signature (CHRNA3, GABRD, GRIK3, GRIK5), which were enriched in cellular metabolic pathways. High expression of these genes was positively correlated with immunosuppressive cell infiltration, and their expression levels in cancer cells significantly affected the response of cancer cells to chemotherapy.

There are also 4 review articles published under this Research Topic. Yang et al. emphasized the cGAS/STING’s crucial role in mediating innate immunity, enhancing interferon release, and influencing TME. STING modulates diverse pathways, including non-innate immune processes like autophagy-dependent ferroptosis, ROS-induced cell death, endoplasmic reticulum stress-mTOR signaling, apoptosis, senescence-associated secretory phenotypes, and cellular metabolism. These effects collectively shape tumor cell progression, highlighting the multifaceted role of cGAS/STING signaling in cancer biology. 2) Zhang et al. discussed content alterations of tumor-educated platelets, including their coding and noncoding RNA, and protein and their role as potential cancer biomarkers in diverse cancer diagnostics. 3) Chen et al. delved into the role of mechanobiology including genetic, biochemical, and mechanical factors and their interplays in cancer progression. Mechanical alterations, such as changes in stiffness and morphology, significantly impact cancer initiation and dissemination. Exploring cancer mechanobiology offers insights for personalized medicine and innovative treatments. Targeting tumor and microenvironment physical properties provides intervention opportunities, aided by advanced imaging and lab-on-a-chip systems for personalized investigations and drug screening. 4) Saxena et al. discussed the crucial role of complement factor H (CFH) as an innate immune checkpoint in cancer control. They also explored molecular functions, interaction with immune cells, clinical implications, therapeutic potential of CFH, and discussed the challenge for CFH as a target in cancer immunotherapy.

In summary, all publications within this Research Topic have improved our understanding of TME remodeling and cancer therapy interplay. Furthermore, these papers may make valuable contributions towards advancing the treatment options available for diverse cancers.
